# Recent Breakthroughs in PET-CT Multimodality Imaging: Innovations and Clinical Impact

**DOI:** 10.3390/bioengineering11121213

**Published:** 2024-11-30

**Authors:** Dildar Hussain, Naseem Abbas, Jawad Khan

**Affiliations:** 1 Department of Artificial Intelligence and Data Science, Sejong University, Seoul 05006, Republic of Korea; hussain.bangash@sejong.ac.kr; 2 Department of Mechanical Engineering, Sejong University, Seoul 05006, Republic of Korea; 3 Department of AI and Software, School of Computing, Gachon University, 1342 Seongnamdaero, Seongnam-si 13120, Republic of Korea

**Keywords:** PET-CT, multimodality imaging, clinical practice, cutting-edge technology innovations, diagnostic imaging, radiotracer development, artificial intelligence, theranostics, patient care

## Abstract

This review presents a detailed examination of the most recent advancements in positron emission tomography–computed tomography (PET-CT) multimodal imaging over the past five years. The fusion of PET and CT technologies has revolutionized medical imaging, offering unprecedented insights into both anatomical structure and functional processes. The analysis delves into key technological innovations, including advancements in image reconstruction, data-driven gating, and time-of-flight capabilities, highlighting their impact on enhancing diagnostic accuracy and clinical outcomes. Illustrative case studies underscore the transformative role of PET-CT in lesion detection, disease characterization, and treatment response evaluation. Additionally, the review explores future prospects and challenges in PET-CT, advocating for the integration and evaluation of emerging technologies to improve patient care. This comprehensive synthesis aims to equip healthcare professionals, researchers, and industry stakeholders with the knowledge and tools necessary to navigate the evolving landscape of PET-CT multimodal imaging.

## 1. Introduction

In the context of unprecedented technological acceleration, the medical imaging landscape has experienced a significant transformation, particularly with the emergence of positron emission tomography–computed tomography (PET-CT) as a pioneering force at the intersection of precision diagnostics and patient-centric care. In recent years, PET-CT has witnessed remarkable advancements, driven by technological innovations and interdisciplinary collaboration. This review article explores the cutting-edge innovations shaping the landscape of PET-CT multimodality imaging, offering a comprehensive overview of the latest developments, challenges, and future directions.

### 1.1. Background

PET and CT are two distinct imaging modalities with complementary strengths. As a radiotracer imaging method, PET utilizes radiotracers labeled with positron-emitting isotopes injected into the body to visualize metabolic processes within the body. As these radiotracers undergo decay, they emit positrons, which annihilate electrons in the surrounding tissues, producing pairs of gamma rays. The PET scanner detects these gamma rays to create functional images highlighting tissues’ metabolic activity. The study by Wadas et al. (2010) [1] highlights the importance of coordinating radiometals of copper, gallium, indium, yttrium, and zirconium for the PET imaging of diseases. It emphasizes the role of radiotracers in PET, which emit positrons and undergo decay to produce gamma rays that are detected by the PET scanner. This research finding underscores the fundamental principle of PET imaging and the significance of radiotracers in capturing metabolic activity within tissues.

Recent advancements in PET systems have contributed to enhanced resolution and image quality, thereby bolstering the utility of PET in both basic science research and clinical imaging. Byrnes et al. (2013) [2] emphasized the technological improvements in PET systems, such as the integration of PET with magnetic resonance imaging (MRI) and the availability of normal healthy human databases and commercial image analysis software. These technological enhancements have led to a growing utilization of molecular imaging in basic science research and clinical imaging, signifying the pivotal role of technological innovations in propelling PET-CT to the forefront of medical research and practice.

On the other hand, CT utilizes X-rays to create detailed cross-sectional images of the body’s anatomy. It is useful to diagnose and monitor a wide variety of conditions, including cancer, trauma, infections, and vascular diseases. CT scans are particularly useful for imaging complex structures such as the brain, chest, and abdomen, as well as for detecting small abnormalities that may not be visible on other types of imaging. However, like X-rays, CT scans use ionizing radiation, which can be harmful in large doses. Therefore, doctors carefully weigh the risks and benefits of CT scans before ordering them and take steps to minimize radiation exposure, such as using low-dose protocols or alternative imaging methods when possible. In recent years, advances in CT technology have led to the development of new imaging techniques, such as cardiac CT angiography and CT colonography, which allow doctors to visualize and diagnose conditions in specific organs or systems of the body [3].

On an individual basis, PET and CT modalities have limitations. PET’s lower spatial resolution may result in reduced anatomical detail, while CT’s reliance on ionizing radiation poses risks. Additionally, PET’s sensitivity to motion artifacts and CT’s limited soft-tissue contrast present challenges. Integrating PET and CT addresses these limitations, enhancing the diagnostic accuracy and clinical utility.

PET-CT imaging is a sophisticated medical imaging technique that combines the strengths of two distinct modalities to provide comprehensive information about both the structure and function of tissues within the body. PET-CT has become an indispensable tool in clinical practice due to its unique ability to merge functional and anatomical data, offering a more holistic understanding of physiological processes and disease pathology. Their integration in a single imaging session has significantly improved diagnostic accuracy, treatment planning, and patient management. This technology continues to evolve, with ongoing research focused on refining the technologies and enhancing the image quality [4]. PET-CT imaging finds widespread applications across various medical specialties, including oncology, neurology, cardiology, and infectious diseases. In oncology, PET-CT plays a pivotal role in cancer staging, treatment response evaluation, and recurrence detection. It aids in the early detection of tumors, guides precision treatment strategies, and facilitates personalized patient care. In neurology, PET-CT contributes to the diagnosis and management of neurological disorders such as Alzheimer’s disease (AD), epilepsy, and brain tumors, providing insights into cerebral metabolism and blood flow [5]. Similarly, in cardiology, PET-CT assists in assessing myocardial perfusion, viability, and function, guiding therapeutic decisions in ischemic heart disease [6]. Crucially, it guides treatment planning, especially in radiation therapy, allowing for precise target delineation, adaptive treatment strategies, and monitoring treatment responses [7,8,9,10,11].

**Figure 1:**
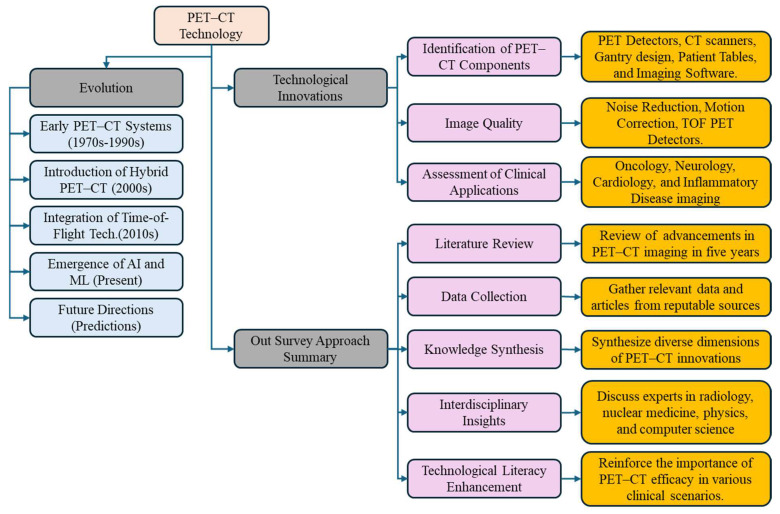
Progression of PET–CT technology from its inception to modern advancements.

The rapid evolution of PET-CT imaging is driven by a convergence of factors, including advancements in detector technologies, image reconstruction algorithms, and artificial intelligence (AI). These advancements not only enhance the diagnostic accuracy but also streamline imaging workflows, enabling clinicians to make informed decisions quickly and effectively. Figure 1 provides a visual representation of the inventory approach used to assess advancements in PET-CT technology, the evolution of PET-CT technology, and summarizing the survey approach, aiding readers in understanding the methodology and its implications for clinical practice and research.

Bailey and Willowson (2014) [12] discussed the advancements in small-animal PET through the development of detector arrays and positron-emitting elements, enabling the labeling of new molecular tracers. They also highlighted the improvements in micro-MRI, particularly in spatial resolution and sensitivity. The ability to image the local mechanical function and solute transport processes is noted, and the synergistic integration of PET with MRI is emphasized. This research insight underscores the potential of PET-CT integration in providing both molecular information and detailed anatomical and functional imaging.

Drzezga et al. (2012) [13] highlighted the clinical significance of PET-CT in patients with oncologic diagnoses, emphasizing its impact on cancer and its crucial role in oncologic clinical practice and translational cancer research. Moreover, Farwell, Pryma, and Mankoff (2014) [14] underscored the current applications of PET-CT imaging in cancer and its potential future directions, signifying the pivotal role of PET-CT in advancing oncologic research and clinical practice. It holds a crucial position in medical research and drug development, providing the non-invasive monitoring of biological processes, aiding in disease mechanism studies, drug testing, and treatment efficacy assessments in trials [15,16]. PET-CT’s sensitivity to metabolic changes facilitates early disease detection, often preceding structural changes observed through other imaging methods [17]. This early detection allows for timely interventions and improved patient outcomes. The fusion of PET and CT data offers a holistic perspective, enabling clinicians to tailor treatment plans based on functional and structural considerations.

Knowledge Gaps and Future Directions: Undeniably, PET-CT has become an indispensable tool in clinical practice, significantly contributing to the diagnosis, treatment planning, and monitoring across a spectrum of medical conditions, making it a cornerstone in modern healthcare. Lecoq et al. (2020) [18] proposed a roadmap for addressing these challenges, emphasizing the need for continued innovation to enhance PET imaging’s temporal resolution. Moreover, future research should prioritize the development of novel technologies and methodologies to overcome current limitations and enhance PET-CT’s clinical utility across diverse medical applications. Despite its clinical utility, PET-CT imaging faces challenges, including radiation exposure, image artifacts, and the need for specialized expertise for interpretation. However, ongoing research and technological advancements are addressing these challenges, with innovations in detector technologies, image reconstruction algorithms, and AI playing a pivotal role in enhancing PET-CT imaging capabilities. From improved spatial resolution to real-time image analysis, these innovations hold immense promise for advancing diagnostic accuracy and patient care in PET-CT imaging. The AI-driven advancements not only expedite interpretation but also contribute to precise lesion segmentation, facilitating quantitative metrics such as standardized uptake values (SUVs) for enhanced diagnosis and treatment response assessment.

### 1.2. Motivation

In recent years, the rapid evolution of PET-CT technology has underscored the necessity for a comprehensive review that synthesizes these advancements. This article seeks to fulfill that need by offering healthcare professionals, researchers, and industry stakeholders a thorough understanding of the current landscape of PET-CT imaging by delving into recent technological breakthroughs.

This review article critically assesses recent developments, pinpointing areas for further exploration and illuminating potential research avenues. By addressing prevalent challenges like image noise and motion artifacts, the article contributes to diverting attention toward the ongoing optimization of PET-CT’s clinical applicability. Notably, a comprehensive literature review can underscore the collaborative nature of PET-CT technology, emphasizing the importance of interdisciplinary cooperation among fields such as radiology, nuclear medicine, physics, and computer science by providing a platform for dialog and knowledge exchange; the review fosters synergy among diverse experts. Moreover, this article contributes to the evidence backing PET-CT’s effectiveness in diverse clinical settings. Technological literacy among healthcare professionals is indispensable, particularly in fields as dynamic and impactful as medical imaging. It empowers us to harness the full potential of innovations in PET-CT, enabling more precise diagnoses and more effective treatment strategies.

Our motivation is clear: by consolidating information, we create a comprehensive understanding of the latest developments in PET-CT technology. Through rigorous analysis, we evaluate their practical implications and potential benefits. And by disseminating this knowledge, we ensure that advancements reach every corner of the healthcare community, driving widespread adoption and optimizing patient care. Ultimately, the driving force behind these technological advancements is our collective commitment to advancing patient care. It is about refining diagnostics to detect diseases earlier and with greater accuracy. It is about pushing the boundaries of medical knowledge to uncover new insights into physiology and pathology. And it is about collaboration—across disciplines, institutions, and borders—to harness the full potential of medical imaging and transform healthcare for the better.

Novel innovations in PET-CT are highlighted in this article regarding multimodalities medical image analysis from the perspective of technological innovations, image quality and quantification, clinical applications, AI integration, challenges, and future directions. This article contributes in several ways regarding PET-CT multimodal medical imaging, as follows:

- This article offers a comprehensive overview of recent advancements in PET-CT, spanning technological innovations, image quality, clinical applications, AI integration, challenges, and future directions.
- It identifies gaps and challenges within the current literature, addressing limitations in existing models and techniques.
- Serving as an accessible educational resource, the review article caters to both researchers and practitioners entering the field of PET-CT imaging.
- By pinpointing gaps, offering best practices, and identifying benchmark datasets, this review aims to steer future research endeavors in PET-CT toward areas with the greatest potential impact.

**Figure 2:**
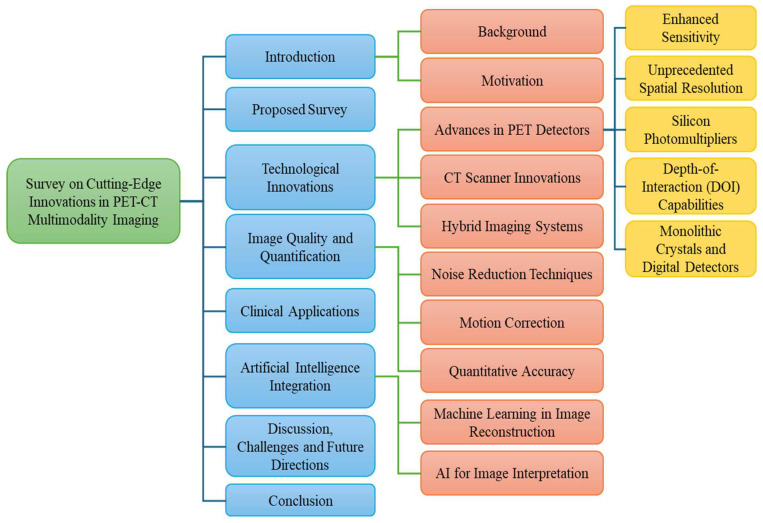
Organization of the survey paper.

These contributions collectively aim to enrich scientific discourse, empower practitioners, and guide future research and technological innovations in the dynamic field of PET-CT multimodality imaging. The survey is organized as follows: Section 2 covers the proposed survey search strategy and study selection criteria. Section 3 reveals the technological innovations in PET-CT. Section 4 concerns the image quality and quantification. Section 5 is about the inventory of clinical applications of PET-CT. Section 6 highlights the integration of AI into PET-CT technology. Section 7 discusses the novel findings of the survey, challenges, and future directions. Finally, Section 8 provides the concluding remarks of this survey. An overview of our survey article is shown in Figure 2.

## 2. Proposed Survey

To comprehensively elucidate the cutting-edge innovations in PET-CT multimodality imaging, a meticulous search strategy and stringent study selection criteria were employed. The primary objective was to ensure the inclusion of relevant and high-quality studies that contribute significantly to the understanding of technological advancements in this field.

### 2.1. Scope of the Review

This review navigates the dynamic landscape of medical innovation, illuminating the recent strides in PET-CT imaging over the past five years. It comprehensively explores various dimensions, delving into the technological breakthroughs enhancing diagnostic precision, from spatial resolution improvements to novel reconstruction algorithms. Beyond diagnostics, the review focuses on PET-CT’s influence on treatment strategies, examining advancements empowering practitioners with personalized intervention information, thus optimizing therapeutic outcomes.

A critical aspect involves assessing real-time treatment efficacy through innovations in PET-CT technology, enabling more sensitive methods for tracking metabolic changes crucial for adapting therapeutic regimens. Addressing intrinsic challenges like image noise and radiation exposure, the review contributes to ongoing efforts to optimize PET-CT’s clinical utility.

The educational scope extends to serving as a resource for students and healthcare professionals navigating the intricacies of PET-CT multimodality imaging. Recognizing collaboration’s essence, the review explores interdisciplinary convergences, fostering dialog among diverse experts from radiology, nuclear medicine, physics, and computer science.

### 2.2. Search Strategy

Database Selection: Utilization of major scientific databases such as PubMed, IEEE Xplore, Scopus, and Google Scholar to ensure a comprehensive retrieval of relevant articles.

Keyword Incorporation: Inclusion of keywords such as “PET-CT innovations”, “multimodality imaging”, “advanced PET-CT technology”, and “clinical applications of PET-CT” to refine the search results.

Boolean Operators: Implementation of Boolean operators (AND, OR) to combine keywords effectively and enhance search precision. Example: (“PET-CT innovations” OR “advanced PET-CT technology”) AND (“multimodality imaging” OR “clinical applications of PET-CT”).

Publication Date: Limiting the search to studies published within the last five years to focus on recent developments and advancements.

Other strategies for searching articles include keywords like “PET-CT imaging”, “Technological innovations in medical imaging”, “Precision diagnostics in medical imaging”, “Patient-centric care in medical imaging”, “Positron Emission Tomography-Computed Tomography advancements”, “PET-CT in medical research”, “PET-CT in clinical practice”, “Medical imaging landscape transformation”, “PET-CT technology trends”, “Recent developments in PET-CT”, etc.

### 2.3. Study Selection Criteria

Relevance to Technological Advancements: The inclusion of studies that explicitly discuss technological innovations in PET-CT, including hardware and software advancements, novel imaging protocols, and integration of AI.

Clinical Impact: Prioritization of studies demonstrating a clear clinical impact of PET-CT innovations on diagnostic precision, treatment planning, and patient outcomes across diverse medical disciplines.

Methodological Rigor: Selection of articles with robust methodologies, including controlled trials, comparative studies, and systematic reviews, to ensure the reliability of reported innovations.

Multidisciplinary Focus: Inclusion of studies showcasing collaborative efforts across radiology, nuclear medicine, physics, and computer science, highlighting the interdisciplinary nature of PET-CT advancements.

Publication Source: Preference for studies published in reputable, peer-reviewed journals and conferences to maintain a high standard of scientific rigor.

Language and Accessibility: Consideration of studies published in English for accessibility, with an emphasis on global representation to capture diverse perspectives.

**Figure 3:**
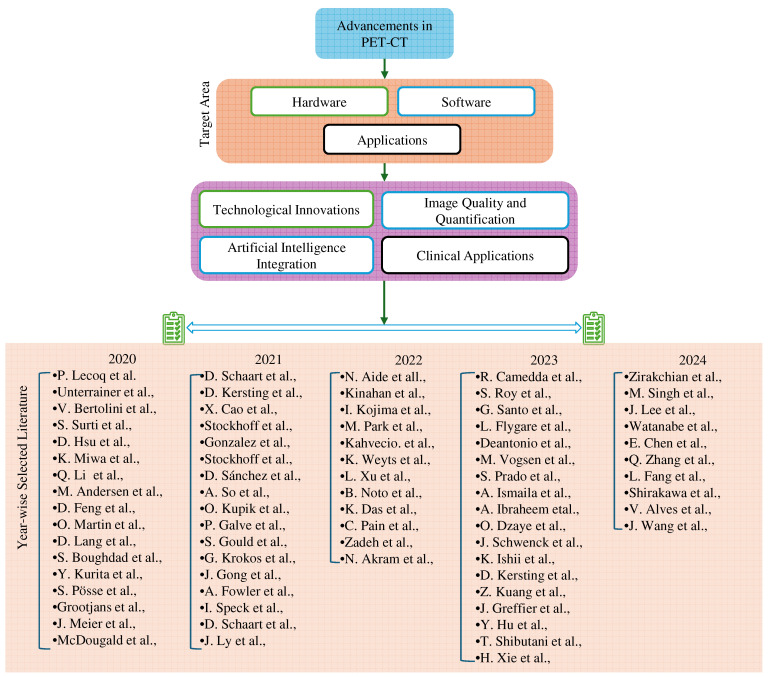
Selected articles with top achievements over the past 5 years focused on PET-CT and multi-model medical imaging modality [5,6,7,8,9,10,11,15,16,17,18,19,20,21,22,23,24,25,26,27,28,29,30,31,32,33,34,35,36,37,38,39,40,41,42,43,44,45,46,47,48,49,50,51,52,53,54,55,56,57,58,59,60,61,62,63,64,65,66,67,68,69,70,71,72,73,74,75,76,77,78,79,80].

**Figure 4:**
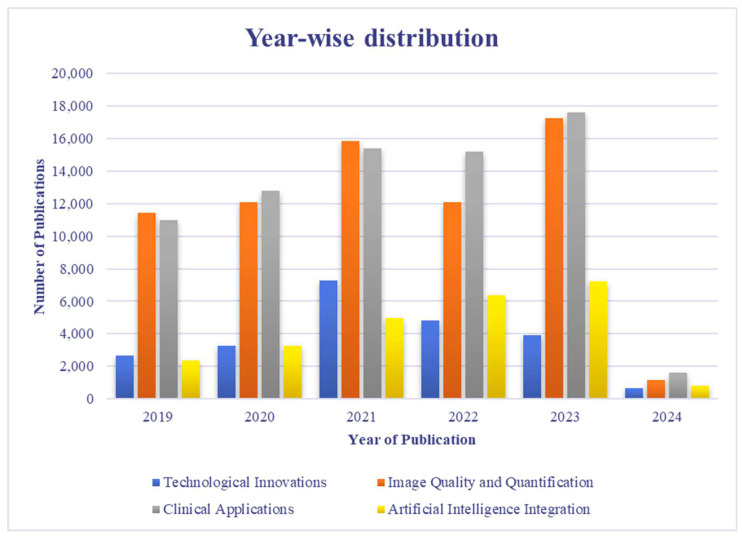
Year-wise distribution of the reviewed literature is based on a Google Scholar query upon all the articles mentioning word, i.e., “CT Scanner Innovations”, “Noise-Reduction Techniques”, and the specific modality, i.e., “PET-CT”. Finally, articles extracted with all of these keywords are accumulated under the main heading, i.e., technical innovations.

**Figure 5:**
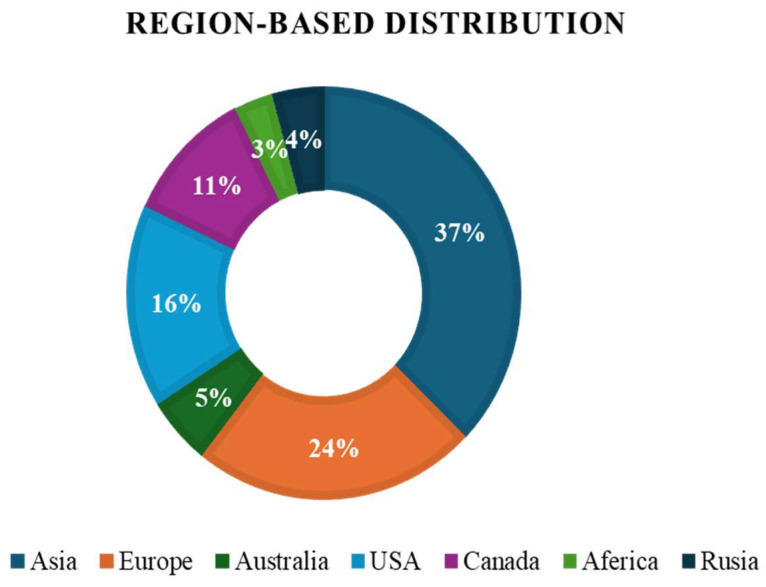
Region-wise distribution of the selected research papers.

**Figure 6:**
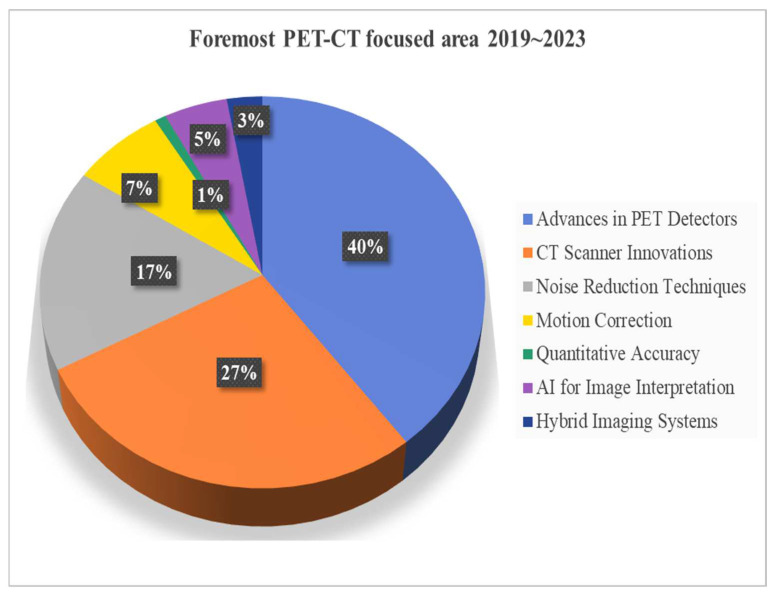
Most focused innovation area distribution of PET-CT.

This comprehensive search strategy and stringent selection criteria aim to provide a nuanced overview of recent innovations in PET-CT multimodality imaging, ensuring the incorporation of impactful studies that significantly contribute to the evolving landscape of clinical practice. Figure 3 shows a year-wise breakdown of selected literature from the past 5 years, high-lighting the top achievements in PET-CT multimodal imaging. Figure 4 shows the year-wise distribution of the chosen literature on the advancement in PET-CT. Figure 5 depicts the most focused innovation area distribution of PET-CT, and Figure 6 shows the region-wise distribution of the selected papers.

### 2.4. Review of Related Surveys

In recent years, several surveys have been conducted to assess the advancements and innovations in PET-CT multimodality imaging technology. These surveys provide valuable insights into the progress made in this field and highlight areas of focus for future research and development, as presented in Table 1. In this review, we aim to build upon the findings of these surveys and provide an updated and comprehensive overview of the latest innovations and breakthroughs in PET-CT imaging.

## 3. Technological Innovations

Technological innovations in PET-CT have established a new era of precision and efficiency in medical imaging. With advancements in detector sensitivity, spatial resolution, and TOF capabilities, contemporary PET-CT systems offer unprecedented clarity in visualizing metabolic and anatomical information. The integration of silicon photomultipliers, digital detectors, and sophisticated image reconstruction algorithms has significantly enhanced diagnostic accuracy. These innovations not only refine cancer detection and staging but also broaden the scope of PET-CT applications in neurology, cardiology, and beyond. The continuous evolution of PET-CT technology underscores its pivotal role in advancing patient care and shaping modern diagnostic practices.

### 3.1. Advances in PET Detectors

PET imaging has undergone significant advancements in detector technologies, marking a transformative era in molecular imaging. This discussion explores the notable progress in PET detectors, specifically focusing on improvements in sensitivity, spatial resolution, and TOF capabilities. Suleman Surti et al., 2020 [25], offered an overview of recent advancements in TOF PET technology, focusing on hardware developments in commercial PET-CT systems. The paper discusses the progress in scintillator technology, photosensors, electronics readouts, and detector designs. The latest PET-CT scanners based on these technologies are introduced, along with insights into their performance. Additionally, it addresses challenges in achieving improved timing and scaling this performance to complete systems. The paper concludes by discussing the outlook for achieving a sub-50 ps coincidence timing resolution (CTR) in PET detectors. Dennis R Schaart et al. (2021) [26] delve into the challenges, solutions, and lessons learned during this period, focusing on detectors based on fast, bright, inorganic scintillators. The review covers the optimization of the coincidence resolving time (CRT) by addressing the entire detection chain and understanding the physics involved. The review examines scintillator and photosensor components, explores the parameters influencing CRT, and discusses current and ongoing developments. While improvements to achieve CRT values around 100 ps seem feasible, reaching ultra-high-precision timestamps may require novel approaches. Ultimately, the goal is to enhance PET’s value in research and personalized medicine.

In the quest for heightened sensitivity, advancements in PET detectors have been monumental. New detector materials, such as lutetium-based crystals like LYSO (lutetium yttrium orthosilicate), have emerged, offering improved light output and timing characteristics [81]. These materials enhance the detector’s ability to capture and measure even faint signals, thus increasing the sensitivity. Additionally, innovations in photodetectors, including silicon photomultipliers (SiPMs) and digital photon counters (DPCs), contribute to enhanced light collection efficiency, further elevating the overall sensitivity of PET detectors [27,28].

In their mini-EXPLORER project, Yang Lv et al. [82] developed a state-of-the-art PET-CT scanner for whole-body imaging, particularly for companion animals and human brain studies. The PET component features high resolution and sensitivity, with a ring diameter of 52 cm and LYSO crystal arrays coupled to SiPMs. The CT component comprises a 24-row scanner with a 50-kW X-ray tube. The PET system parameters include a TOF resolution of 409 ± 39 ps, an energy resolution of 11.7% ± 1.5%, and a system sensitivity of 52–54 kcps MBq−1. The spatial resolution was 2.6 mm at 10 mm from the center, with the successful imaging of a brain phantom and a canine patient. The CT scanner met the ACR accreditation standards.

Spatial resolution is a critical determinant of image quality in PET imaging. Recent developments have seen a remarkable improvement in spatial resolution, allowing for more precise localization of radiotracer uptake. Silicon photomultiplier technology, characterized by its compact size and excellent timing properties, is pivotal in achieving a finer spatial resolution [83]. Kazunari Ishii et al. (2023) [29] investigated and compared brain PET images acquired using a dedicated head PET system (dhPET) with a conventional whole-body PET-CT (wbPET) system. Visual inspection, voxel-wise analysis, and the SUV ratio (SUVR) were utilized. DhPET images showed superior visual quality and higher SUVRs compared to wbPET. Regional accumulation differences were observed, suggesting the potential benefits of dhPET in clinical practice. Understanding these differences will optimize the utilization of dhPET in diagnostic imaging. Additionally, integrating advanced image reconstruction algorithms, such as TOF reconstruction, contributes to sharpening the spatial resolution by leveraging the temporal information of photon arrivals.

TOF capabilities represent a revolutionary stride in PET imaging. PET scanners equipped with TOF capabilities enhance the localization accuracy by measuring the time it takes for photons to travel from the emission source to the detector. This temporal information allows for a more precise determination of the origin of annihilation events, resulting in an improved spatial resolution. Recent TOF advancements include faster scintillation materials, optimized electronics, and sophisticated algorithms that exploit temporal data to create images with unprecedented clarity. Kenta Miwa et al. (2020) [30] evaluated the performance of a new digital TOF-PET-CT scanner with a Bayesian penalized likelihood (BPL) PET reconstruction algorithm (Q.Clear) in detecting small lesions. PET data from a NEMA body phantom were reconstructed using various algorithms and acquisition times. The results showed an improved recovery coefficient (RC) and detectability index (DI) for sub-centimeter spheres with BPL reconstruction compared to other methods. However, BPL led to uptake overestimation in 8-mm spheres due to edge artifacts. Optimal performance was observed with a β value of 200 for detecting sub-centimeter lesions, highlighting the potential of BPL in enhancing lesion detection in clinical practice.

The integration of SiPMs has been a game-changer in PET detector technology. These solid-state photodetectors offer advantages such as a compact size, low power consumption, and excellent timing resolution. SiPMs have replaced traditional photomultiplier tubes (PMTs) in some PET systems, improving the detector efficiency and advancing the spatial and temporal resolutions [31,32,33,34,35].

Addressing the challenge of parallax errors, Depth-of-Interaction (DOI) capabilities have gained prominence in PET detector design. These capabilities enable the system to distinguish between annihilation events occurring at different depths within the detector crystal, providing more accurate localization information. Combined with advancements in crystal design and readout technology, DOI capabilities contribute to improved overall image quality [36,37,38,84].

The use of monolithic crystals—single, continuous crystals without gaps—has become increasingly prevalent. Monolithic crystals enhance the spatial resolution by minimizing the spatial uncertainties that crystal segmentation introduces. Digital detector technology, including DPCs, facilitates direct digital readout, eliminating the need for analog signal processing and further improving the detector performance [39,40,41].

### 3.2. CT Scanner Innovations

The ever-evolving landscape of CT scanner technologies has witnessed transformative innovations, with two key developments, spectral CT and iterative reconstruction algorithms, at the forefront of this paradigm shift. Within the broader context of technological innovations in PET-CT, CT scanner advancements are a pivotal domain reshaping the medical imaging landscape. Notably, the introduction of spectral CT marks a revolutionary stride. This innovation enables the simultaneous acquisition of multiple energy datasets, unlocking unparalleled tissue characterization and material differentiation capabilities. Spectral CT expands the diagnostic horizon, which is particularly transformative in fields like oncology and vascular imaging [42,43,44].

Spectral CT represents a monumental leap forward in diagnostic capabilities. This innovation allows for the simultaneous acquisition of multiple energy datasets during a scan, offering a comprehensive view of the tissue composition. Unlike conventional CT, spectral CT enables enhanced tissue characterization and material differentiation. This proves invaluable, especially in complex clinical scenarios such as oncology, where the precise identification of different tissue types is critical. The ability to discern subtle differences in contrast and composition enhances the diagnostic accuracy and opens new possibilities for personalized and targeted treatment strategies. Osman Kupik et al. (2021) [45] assessed the correlation between dual-energy spectral computed tomography (DESCT) and 18F-fluorodeoxyglucose (FDG) PET-CT parameters in lung cancer patients. However, no significant correlation was found between the DESCT and PET-CT parameters in primary tumors or metastatic lymph nodes, suggesting that these modalities represent distinct tumor characteristics and should not replace each other in clinical practice. Michael Brun Andersen et al. (2020) [46] evaluated the diagnostic performance of the spectral dual-layer detector CT compared to the conventional contrast-enhanced CT (CE-CT) in detecting and characterizing cancer. The results showed that spectral CT identified significantly more cancer findings with a higher sensitivity (89% vs. 77%) and slightly lower specificity (77% vs. 83%) compared to conventional CT. Additionally, spectral CT increased the diagnostic certainty for cystic lesions, particularly in the kidney, liver, thyroid, and ovaries, leading to a reduction in the need for follow-up examinations.

Iterative reconstruction algorithms are pivotal in addressing the perennial challenge of balancing the image quality and radiation dose. By refining the image reconstruction process, these algorithms enhance the clarity and precision of CT scans while minimizing patients’ exposure to ionizing radiation. The iterative approach involves multiple cycles of refining the image based on the comparison with the acquired data, resulting in clearer and more detailed imaging. This contributes to more accurate diagnoses and aligns with the imperative of patient safety, a paramount consideration in modern healthcare [47,48,49].

Yan Hu et al. (2023) [50] assessed the feasibility of using ultra-low-dose CT (ULDCT) reconstructed with an AI iterative reconstruction (AIIR) algorithm in total-body PET-CT imaging. The results showed that ULDCT reconstructed with AIIR provided image quality comparable to standard-dose CT (SDCT) reconstructed with hybrid iterative reconstruction (HIR), with decreased image noise and an increased signal-to-noise ratio (SNR). While ULDCT-AIIR could not entirely replace SDCT-HIR, it could be considered in specific situations for PET-CT examinations, offering potential benefits in reducing radiation exposure without compromising the diagnostic accuracy.

Ew-Jun Chen et al. (2024) [51] highlighted the evaluation of a novel reconstruction algorithm, HYPER DPR, compared to the conventional Ordered Subset Expectation Maximization (OSEM) algorithm in PET-CT. The results indicate that HYPER DPR, particularly in digital PET-CT, offers improved spatial resolution, sensitivity, and reduced image noise, enhancing small lesion detection and disease detection, potentially allowing for lower radiation doses and improved patient management.

The synergy between spectral CT and iterative reconstruction algorithms amplifies their individual merits. The enhanced tissue characterization from spectral CT, combined with the optimized image quality and reduced radiation dose achieved through iterative reconstruction, represents a significant leap forward in diagnostic imaging. This collective impact extends across various medical specialties, from oncology and cardiology to neurology and beyond, providing clinicians with a more nuanced and detailed understanding of the patient’s anatomy and pathology.

### 3.3. Hybrid Imaging Systems

The dynamic field of PET-CT has experienced groundbreaking advancements, reshaping the landscape of hybrid imaging. These novel developments go beyond the traditional boundaries, ushering in a new era of precision and versatility in diagnostic capabilities.

Novel PET-CT systems now leverage simultaneous acquisition technology, enabling the concurrent capture of PET and CT images. This approach, facilitated by cutting-edge detector technologies, ensures perfect anatomical and functional alignment. Integrating TOF capabilities and advanced scintillation materials enhances the sensitivity and spatial resolution. Simultaneous acquisitions streamline the imaging process, reducing scan times and eliminating potential misalignments between PET and CT datasets, offering a comprehensive view of functional and anatomical aspects in real-time [52,53].

David Dagan Feng et al., 2020 [54], underscored the recent advancements in PET-CT technology, particularly in enabling the non-invasive absolute quantification of physiological function through the accurate estimation of the plasma time–activity curve (PTAC). The research highlighted the evolution of PTAC estimation methods, discussed recent insights from the uEXPLORER total-body PET-CT device, and emphasized the potential of the kinetic model-based simultaneous estimation of input functions and physiological parameters (SIME) method for enhancing the quantification quality in PET studies. Overall, these advancements hold promise for advancing future PET research by providing more reliable and precise quantitative data.

In contrast to simultaneous acquisitions, sequential acquisition strategies involve separate PET and CT scans conducted in sequence. This provides flexibility in imaging protocols, allowing for the distinct optimization of PET and CT parameters based on clinical requirements. Sequential acquisitions are particularly advantageous in scenarios where specific imaging protocols are essential, offering tailored solutions for diverse diagnostic needs.

Ole Martin et al. (2020) [55] compared PET–magnetic resonance imaging (MRI) and PET-CT in lesion detection and classification in oncologic whole-body examinations while investigating radiation exposure differences between the two modalities. The results showed that PET-MRI provided additional information in a significant percentage of cases, leading to changes in TNM staging and improved lesion detectability in selected cancers. PET-MRI also demonstrated the potential for reducing radiation exposure compared to PET-CT, which is particularly beneficial for younger patients. Overall, PET-MRI offers comparable staging capabilities to PET-CT, with the added advantages of enhanced lesion detection and reduced radiation exposure. According to Dong Zheng et al. (2020) [85], PET-CT and PET-MR imaging were sequentially performed in 30 patients using a single-injection-with-dual-imaging protocol. The results showed that PET/MR with the T2-weighted half-Fourier acquisition single-shot turbo spin-echo (T2W-HASTE) sequence provided superior image quality and lesion conspicuity compared to PET-CT, indicating the potential of PET-MR with T2W-HASTE for diagnosing and staging gastric cancer.

A. Kahvecioglu et al. (2022) [56] evaluated both simultaneous integrated boost (SIB) and sequential boost (SB) approaches for clinically positive lymph nodes (LNs) in locally advanced cervical cancer (LACC). Both approaches demonstrated excellent local control rates for boosted LNs with a low toxicity. Therefore, the study did not identify a clear superiority between SIB and SB, suggesting that both approaches are effective options for treating involved LNs in LACC.

The clinical impact of these novel PET-CT advancements is profound. Simultaneous acquisitions excel in dynamic studies, such as cardiac imaging, where precise alignment is crucial for accurate functional assessments. Sequential acquisitions find utility in scenarios demanding distinct optimization, such as oncological studies requiring specific PET and CT protocols. Clinicians benefit from a more comprehensive and nuanced understanding of pathology, enabling targeted interventions and personalized treatment strategies [57,58,59].

The synergy between PET and CT modalities, enhanced by these advancements, underscores the holistic nature of hybrid imaging. The seamless integration of metabolic insights from PET and detailed anatomical information from CT refines the diagnostic accuracy. This is particularly valuable in fields like oncology, neurology, and cardiology, where a comprehensive understanding of both functional and structural aspects is essential for optimal patient care. Technological innovations are summarized in Table 2.

## 4. Image Quality and Quantification

Pursuing superior image quality and precise quantification is the cornerstone of PET-CT evolution. Striking the delicate balance between spatial resolution and sensitivity refines lesion detection, fostering the diagnostic accuracy. Innovations in reconstruction algorithms enhance both structural and functional insights, advancing clinical decision making. Quantitative accuracy, particularly in SUVs, undergoes meticulous scrutiny, with efforts to standardize measurements across diverse systems. This relentless pursuit of image fidelity and quantification reliability underscores PET-CT’s commitment to delivering unparalleled precision in depicting the intricacies of physiological processes, propelling it to the forefront of modern medical imaging. These innovations are summarized in Table 3.

### 4.1. Noise-Reduction Techniques

In the realm of PET-CT imaging, the presence of noise can compromise the clarity and accuracy of the diagnostic information. As such, the development of effective noise-reduction techniques is pivotal for optimizing the image quality and bolstering the precision of clinical assessments. This exploration delves into the various methods employed to mitigate noise in PET-CT images [60,61,67].

Advanced image reconstruction algorithms play a central role in noise reduction. Iterative reconstruction techniques, such as OSEM, leverage multiple iterations to refine the image quality. These algorithms contribute to smoothing out irregularities and reducing noise, resulting in clearer and more diagnostically relevant images. In their study, Yuya Shirakawa et al. (2024) [68] assessed the quantitative values of PET images using a silicon photomultiplier (SiPM)-PET-CT system with both phantom and clinical images. The evaluation included the SUV, percent contrast (QH), coefficient of variation in the background area (CV background), and the SNR of liver lesions in clinical imaging. The results showed that the clear adaptive low-noise method (CaLM) yielded higher SUVmax values, improved the contrast, and increased the SNR compared to a 4-mm Gaussian filter (GF), suggesting CaLM’s potential suitability for diagnosis in clinical settings.

Incorporating TOF technology in PET-CT scanners enhances the temporal resolution. TOF imaging improves the SNR by measuring the time taken for emitted positrons to reach the detectors. This refinement accelerates image acquisition and diminishes the impact of random events, reducing noise and enhancing the overall image quality [65,69].

Gaussian filtering is a spatial domain technique that involves applying a convolution kernel to the image. This method effectively suppresses high-frequency noise while preserving essential image details. By adjusting the filter parameters, radiologists can tailor the level of noise reduction according to specific diagnostic requirements [62].

Subsampling involves acquiring fewer data points during the PET-CT scan, followed by interpolation to reconstruct a complete image. While this may reduce noise, it requires careful consideration to maintain the diagnostic accuracy. Advanced subsampling algorithms aim to strike a balance between noise reduction and preserving crucial imaging information [63].

Post-processing filters applied to reconstructed PET-CT images offer an additional layer of noise reduction. These filters, such as bilateral filters, selectively smooth image regions while preserving edges and structures. By targeting noise without compromising essential details, post-processing techniques contribute to an overall improvement in image quality [70,71].

This sophisticated approach combines statistical principles with regularization techniques. Bayesian penalized likelihood reconstruction incorporates prior knowledge about expected image characteristics, enabling a more nuanced noise reduction. The result balances noise suppression and the preservation of clinically relevant details [64,72,73,93,94].

### 4.2. Motion Correction

Motion artifacts pose a significant challenge in PET-CT imaging, potentially compromising the accuracy of clinical assessments. Implementing effective strategies for motion correction is paramount to ensure the fidelity of dynamic imaging and to address issues arising from respiratory motion. This discussion explores the various approaches employed to mitigate motion artifacts in PET-CT [86,95].

L.K.S. Sundar et al. (2023) [96] introduced Fast Algorithm for Motion Correction (FALCON) software for correcting both rigid and nonlinear motion artifacts in dynamic whole-body (WB) PET-CT images. FALCON utilizes multiscale image alignment and automated frame selection to achieve accurate motion correction. Evaluation across different PET-CT systems and tracers demonstrated a significant reduction in motion artifacts, improved image quality, and the preservation of activity concentration levels, making FALCON suitable for various PET imaging scenarios.

Respiratory gating involves synchronizing the PET-CT acquisition with the patient’s respiratory cycle. By dividing the respiratory motion into specific phases, images can be acquired during periods of reduced motion, thereby minimizing blurring. This method enhances the precision of imaging, particularly in areas affected by respiratory motion, such as the thoracic region [87,97].

Amplitude gating focuses on capturing images during the specific amplitude ranges of respiratory motion. By selecting optimal phases based on amplitude, this approach targets periods of minimal motion, reducing artifacts associated with respiratory variations. Amplitude gating is particularly effective in regions prone to respiratory-induced motion, offering improved image quality [74,98].

A study by Willem Grootjans et al. (2020) [75] emphasizes the significance of correcting respiratory motion artifacts in the PET-CT imaging of the thorax and upper abdomen to enhance the image quality and quantitative accuracy. It introduces an amplitude-based optimal respiratory gating (ORG) algorithm, providing users with control over balancing image quality and motion rejection by adjusting the duty cycle. By effectively removing respiratory motion-induced blurring, ORG improves the quality and accuracy of PET-CT images, thereby facilitating the more precise diagnosis and staging of various diseases.

List-mode data reconstruction allows for the retrospective selection of data during post-processing. This flexibility enables the identification and exclusion of motion-affected data, resulting in clearer images. List-mode reconstruction is particularly advantageous in dynamic imaging scenarios, where precise temporal alignment is critical [99].

Advanced motion-compensated image reconstruction algorithms account for motion-induced discrepancies during the reconstruction process. These algorithms utilize motion models derived from acquired data to correct motion artefacts, providing enhanced image quality and diagnostic accuracy [88].

Real-time monitoring devices track respiratory motion throughout the PET-CT scan. These devices, often based on external surrogates or internal fiducial markers, provide continuous feedback to enable the correlation of acquired images with specific respiratory phases. This ensures accurate motion correction and improves the overall quality of PET-CT imaging [66,76].

Dedicated motion-tracking software monitors and corrects involuntary patient movements during the scan. This includes respiratory motion and other sources of motion, such as patient coughing or body shifts. Real-time adjustments based on continuous tracking contribute to motion artifact reduction [100,101].

W. Tan et al. (2023) [102] addressed patient motion issues in the PET imaging of the human brain by employing a wearable electromagnetic motion tracking system for the precise real-time estimation of head position and orientation. The research evaluated the performance of PET-CT head motion correction using this system and proposed an automatic intersystem calibration method to streamline the coordination between the motion tracking and PET scanner systems, aiming to enhance the image quality and quantification accuracy.

### 4.3. Quantitative Accuracy

Quantitative accuracy, particularly in SUV measurements, is a critical aspect of PET-CT imaging. Improvements in this domain have a direct impact on the precision of clinical assessments and play a pivotal role in guiding treatment decisions. This exploration delves into the strides made in enhancing the quantitative accuracy, with a specific focus on refining SUV measurements. Wendy McDougald et al. (2020) [77] evaluated the variability of current preclinical PET-CT acquisition and reconstruction protocols across multiple centers and scanners, and proposed standardized protocols for multicenter data. Five different commercial preclinical PET-CT scanners were assessed, revealing significant differences in CT and PET parameters among sites. By implementing standardized protocols, improvements in PET-CT accuracy and precision were observed, along with a reduced CT absorbed dose across sites, facilitating reproducible and consistent preclinical imaging datasets and enhancing translational research.

Point Spread Function (PSF) reconstruction algorithms address the inherent blurring in PET images, especially at the edges of structures. By modeling the spread of positron annihilation events more accurately, PSF reconstruction mitigates partial volume effects, resulting in sharper and more quantitatively accurate SUV measurements. This is particularly advantageous in small lesions and regions with high metabolic activity [89].

Accurate respiratory motion correction techniques contribute to improved SUV measurements. Strategies such as respiratory gating and motion-compensated reconstruction ensure that the SUV values are not compromised by motion artifacts. This is crucial, especially in thoracic and abdominal imaging, where respiratory motion can significantly impact quantitative accuracy [90].

Iterative reconstruction algorithms, such as AI and TOF iterative reconstruction, incorporate advanced mathematical models to enhance the image quality and quantitative accuracy. These algorithms iteratively refine the reconstruction process, considering factors like scatter and attenuation correction, leading to more reliable SUV measurements [103]. Dirk Hellwig et al. (2023) [104] explored the potential of AI and deep learning (DL) for improving PET image reconstruction, highlighting advancements in resolution, noise reduction, and artifact removal. Challenges such as data availability and cross-scanner compatibility are discussed, along with the foreseeable clinical impact and future trends in integrating AI-based reconstruction into routine PET imaging protocols.

Harmonization efforts and standardization protocols aim to minimize the variability in SUV measurements across different PET-CT systems and institutions. Initiatives such as the EARL (EANM Research Ltd.) accreditation program provide guidelines for calibration and quality control, fostering consistency in quantitative measurements and facilitating multicenter research [91,92].

Advancements in scanner calibration methods contribute to enhanced quantitative accuracy. The regular and precise calibration of PET-CT scanners using standardized phantoms and procedures ensures that SUV measurements are traceable, reproducible, and reliable across different imaging sessions and systems. Edwin K Leung et al. (2021) [105] studied scanner calibration and comprehensively evaluated the quantitative accuracy of the uEXPLORER total-body PET scanner across various imaging applications, demonstrating a relative count rate accuracy of ±3–4%, an axial uniformity spread of ±3%, and a 3% activity bias spread. Region-of-interest quantification showed a spread of 1% and a stable volume-of-interest quantification across a wide range of counts. Overall, the uEXPLORER scanner exhibited uniform quantitative performance across its axial field of view, supporting diverse imaging applications with accuracy.

## 5. Clinical Applications

The integration of PET-CT has brought about a transformative paradigm shift in clinical practice across diverse medical disciplines. This section delves into the myriad clinical applications of PET-CT, highlighting its instrumental role in shaping diagnostic, therapeutic, and monitoring approaches. A summary of clinical practice is presented in Table 4.

PET-CT stands as a cornerstone in oncology, offering unparalleled precision in cancer diagnostics. From characterizing lesions to determining the extent of cancer spread, it plays a pivotal role in accurate staging. Traditional methods like ultrasound, CT, and MRI have limitations in diagnosing cancer. However, recent advancements in hybrid PET-CT and PET-MRI systems have been introduced as transformative tools in cancer diagnosis and treatment. Moreover, in cancer treatment planning, particularly in radiation therapy, PET-CT’s ability to delineate target volumes allows for adaptive and personalized treatment strategies [106]. Anita Brink et al., 2023 [107], explored the role of PET-CT in pediatric oncology, emphasizing its utility in accurately staging disease and assessing metabolic response to treatment. Specifically, their research discusses the use of 18F-FDG PET-CT in lymphomas, neuroblastomas, sarcomas, and Langerhans-cell Histiocytosis (LCH), outlining the considerations, strengths, weaknesses, and current recommendations for each disease entity. The paper underscores the growing importance of PET-CT in guiding treatment decisions and highlights potential future developments in this field.

In neurology, PET-CT imaging offers valuable insights beyond the scope of merely identifying structural abnormalities observable through conventional imaging modalities like MRI and CT scans. It offers functional insights into the brain activity, metabolism, and blood flow, which are essential for understanding various neurological conditions. A prominent instance is AD, in which PET-CT scans can identify alterations in the glucose metabolism and the existence of amyloid plaques in the brain, contributing to the timely detection and continuous monitoring of the disease. Similarly, in epilepsy, PET-CT helps to identify regions of abnormal brain activity, guiding treatment decisions such as surgical intervention. Additionally, PET-CT imaging has demonstrated its indispensability in the evaluation of brain tumors, enabling the differentiation between benign and malignant lesions based on their metabolic features. Ultimately, the utilization of PET-CT imaging has markedly enhanced the precision of the diagnosis and treatment of neurological conditions by furnishing comprehensive functional data associated with the brain [108,109,110,111].

In the domain of cardiology, PET-CT plays a crucial role in the assessment of myocardial perfusion, vitality, and function, offering a fundamental insight into cardiac well-being. Through the identification of regions with diminished blood flow, PET-CT aids in the detection of ischemic heart disease and the evaluation of myocardial injury extent. This information is indispensable for informing treatment decisions, which may involve the consideration of revascularization interventions like angioplasty or coronary artery bypass graft surgery. Additionally, PET-CT enables the evaluation of myocardial viability, helping clinicians to determine whether areas of the heart affected by ischemia can recover with appropriate intervention. PET-CT imaging enhances the diagnostic accuracy and management of cardiovascular conditions, contributing to improved patient outcomes in cardiology practice [112,113,114,115].

This technology plays a crucial role in localizing areas of infection and inflammation, offering valuable insights into various pathologies. Its ability to differentiate infectious processes from other conditions aids in accurate diagnoses and treatment planning [11,20,116]. Additionally, it enables clinicians to monitor the effectiveness of treatment interventions by assessing changes in metabolic activity over time. The concept of theranostics, integrating diagnostic imaging with targeted therapy, finds its embodiment in PET-CT. This capability guides therapeutic decisions and helps to optimize patient care. PET-CT’s utility in infection and inflammation extends beyond diagnosis, contributing to more precise and personalized management and targeted treatment plans based on specific disease characteristics for patients [117,118].

**Table 4:** Summary of clinical innovations in PET-CT.

Article	Year	Target Area	Achievements
M.S. Kalemaki et al., [106]	2020	Ophthalmic Oncology	Hybrid PET-CT and PET-MRI improve orbital and ocular tumor management through early detection, accurate staging, and personalized treatment strategies.
Anita Brink et al., [107]	2023	Pediatric Oncology	Discussed PET-CT in pediatric oncology, emphasizing its role in staging, treatment guidance, and assessing metabolic response, particularly in lymphomas, neuroblastomas, sarcomas, and LCH.
E.M. Teichner et al., [108]	2023	Neurology	PET-CT with NaF showed carotid microcalcification, while FDG-PET indicated brain hypometabolism, suggesting cerebrovascular involvement. PET tracers like NaF aid in the diagnostic accuracy.
R. Camedda et al., [5]	2023	Neurology/Alzheimer’s disease	Correlating cerebrospinal (CSF) dopamine transporter (DAT) levels with the regional brain glucose metabolism in AD patients revealed positive correlations, suggesting a potentially less-advanced disease status.
M. Dietz et al., [112]	2023	Cardiology	Reduced global stress myocardial blood flow (MBF) emerged as the strongest predictor of major adverse cardiovascular events (MACEs), surpassing the global myocardial flow reserve (MFR) and regional severely reduced the myocardial flow capacity (MFCsevere).
Kwiecinski et al., [113]	2023	Cardiology	PET and CTA advancements enable the non-invasive assessment of coronary plaque inflammation and calcification, enhancing risk stratification and improving coronary artery disease management.
M. Vogsen et al., [11]	2023	Breast	Compared CE-CT and 18F-FDG PET-CT for monitoring metastatic breast cancer treatment response. The 18F-FDG PET-CT detected progression earlier in most patients, potentially delaying progression detection by CE-CT by 6 months. PERCIST identified measurable disease in more patients than RECIST 1.1.
J.M.S. Sohns et al., [116]	2020	Cardiology	Whole-body 18F-FDG PET-CT effectively assesses the left-ventricular assist device (LVAD) infection extent, predicts outcomes, and guides early surgical intervention, potentially improving patient care.
L. Filippi et al., [117]	2023	Prostate Cancer	Theranostics integrates diagnosis, therapy, and monitoring. Advances in PSMA and FAPI-PET for prostate cancer, alongside digital and total-body PET-CT, revolutionize oncology.
Y.L. Xie et al., [119]	2021	Tuberculosis (TB)	In TB drug development, [18F]FDG-PET-CT imaging complements sputum bacterial counts, revealing drug efficacy and lesion-specific responses, guiding new regimen evaluation.
R.D. Seban et al., [120]	2021	Spleen	Prognostic value of spleen glucose metabolism on [18F]-FDG PET-CT elucidated across diverse cancers, informing potential future cancer management strategies.

The integrated technology of PET-CT plays a crucial role not only in clinical environments but also in medical research and the progression of pharmaceuticals, i.e., drug/medicinal development. By conducting the non-invasive monitoring of biological processes, PET-CT enables researchers to meticulously analyze the origins of diseases, assess novel medications, and gauge the efficacy of interventions in both experimental and clinical trials. This capability enriches the comprehension of illnesses and the development of comprehensive therapies, consequently bolstering patient outcomes and the pool of medical insights [119,120,121,122].

PET-CT’s sensitivity to metabolic changes enables the early detection of diseases, often preceding structural changes observed through other imaging modalities. The combination of PET and CT data provides a holistic view of the patient’s condition, facilitating more informed clinical decision making. In essence, the clinical applications of PET-CT are vast and diverse, making it an indispensable asset in modern healthcare. From oncology to neurology, cardiology, and beyond, its multifaceted utility revolutionizes clinical practice, offering new dimensions in precision medicine and patient care.

**Benchmark Datasets:** Benchmark datasets play a pivotal role in evaluating the advancement of PET-CT models for multimodality imaging. These datasets, meticulously curated to represent diverse clinical scenarios and imaging challenges, serve as standardized reference points for assessing the performance and robustness of novel PET-CT algorithms and methodologies. By providing a common ground for comparison, benchmark datasets enable researchers to objectively measure the efficacy, accuracy, and generalizability of their models across different imaging modalities, patient populations, and disease presentations. The utilization of these datasets for this specific objective has been comprehensively explained and expounded upon in Table 5.

## 6. Artificial Intelligence Integration

ML algorithms in PET-CT image reconstruction represent a paradigm shift, ushering in unprecedented improvements in both speed and accuracy. This review explores the transformative impact of ML on image reconstruction, highlighting key advancements and their implications for clinical practice. Table 6 describes details about AI-based advancement in PET-CT.

The development of DL-based computer-aided diagnostic systems (CADs) and treatment planning represents a revolutionary advancement in medical imaging. DL holds the potential to reduce the workload for healthcare personnel while simultaneously improving patient outcomes. Our previous research in ML and DL, published in esteemed journals, extensively explored these techniques in diverse domains such as computer vision, natural language processing, medical image analysis, segmentation, and classification [80,131,132,133,134,135,136,137,138,139,140,141,142]. Building upon this foundation, our current focus extends to exploring the enhancement of the accuracy and efficiency in PET-CT. This involves the application of DL in automated medical image analysis, computer-aided diagnosis, and prediction, all of which contribute to the ongoing evolution of PET-CT in healthcare.

The integration of AI into PET-CT has brought a transformative era in medical imaging, i.e., evolving algorithms, sophisticated in nature, amplify the diagnostic accuracy and streamline image reconstruction processes. Beyond mere automation, AI augments clinical decision making by uncovering intricate patterns and nuances in PET-CT scans. The marriage of ML and image interpretation enhances the efficiency and paves the way for personalized patient assessments. As AI becomes an indispensable ally, the synergy between human expertise and computational insights propels PET-CT into a realm where precision meets innovation, redefining the landscape of diagnostic imaging with unprecedented clarity. The advent of AI has brought about transformative changes in PET-CT image interpretation, particularly in automating crucial tasks such as lesion detection, segmentation, and diagnostic decision support. This exploration delves into the multifaceted role of AI in reshaping the landscape of image interpretation, offering unprecedented efficiency and accuracy [123,124].

In order to automate the detection of lesions in PET-CT images, AI algorithms have demonstrated remarkable prowess. By leveraging intricate patterns within the data, these algorithms excel at identifying regions of abnormal metabolic activity indicative of pathological conditions. Automated lesion detection and segmentation with remarkable accuracy significantly expedites the interpretation process, enabling radiologists to focus on nuanced analyses [125,130]. Precise lesion segmentation is crucial for obtaining quantitative metrics, such as SUVs, aiding in both diagnosis and treatment response assessment, which enhances the reproducibility of measurements and provides a more detailed characterization of lesions [126,127]. Serving as a powerful ally in diagnostic decision support, AI offers insights that complement the expertise of radiologists.

In the realm of the PET-CT imaging database/record, AI algorithms play a pivotal role in analyzing extensive datasets to discern nuanced patterns associated with various diseases. By doing so, they offer clinicians valuable insights for informed decision making in patient management and treatment planning. Furthermore, AI extends the scope of image interpretation beyond imaging data by integrating clinical information like patient history and laboratory results. This holistic approach enhances the diagnostic accuracy and fosters personalized, patient-centered care within the context of PET-CT imaging [78,128,129,143].

In their study, Zirakchian Zadeh et al. (2022) [79] evaluated the utility of pre-ablation real-time split-dose PET combined with non-contrast CT in detecting colorectal liver metastasis (CLM) for ablation and assessing ablation zone margins. The findings showed that PET-CT significantly improved CLM detection and margin assessments compared to CT alone, with only a small fraction of CLMs remaining undetectable and a low FDG avidity on PET-CT. This approach offers a valuable alternative to repeated contrast administration, providing the continuous visualization of FDG-avid CLMs during ablation procedures.

By exhibiting continuous learning capabilities, AI systems refine their performance over time. Through exposure to diverse datasets and real-world scenarios, these algorithms adapt to variations in imaging patterns and emerging medical knowledge. This adaptability ensures that AI-driven interpretations remain relevant and effective in evolving healthcare landscapes. While AI holds immense promise, challenges such as algorithm robustness, interpretability, and ethical considerations require careful attention. Ensuring the transparency of AI models, addressing biases, and adhering to rigorous validation processes are essential for the responsible integration of AI into clinical practice.

## 7. Discussion, Challenges, and Future Directions

The present study delves into the transformative impact of cutting-edge innovations in PET-CT multimodality imaging on clinical practice. The following key points emerged from our investigation:

PET-CT technology has rapidly advanced, enhancing the sensitivity, spatial resolution, and TOF capabilities, leading to improved diagnostic accuracy and patient care. Implementing noise-reduction techniques improves the image quality, empowering clinicians with clearer PET-CT images for more precise diagnoses. Strategies like respiratory gating and motion-compensated reconstruction ensure high-quality images, supporting accurate diagnoses and treatment decisions, especially in dynamic scenarios. Technological advancements, including TOF technology and AI integration, elevate the precision of quantitative assessments, reinforcing PET-CT’s reliability in clinical practice and research. PET-CT finds diverse applications beyond oncology, extending into neurology, cardiology, and inflammatory diseases, enhancing multidisciplinary medical practices. Emphasizing a patient-centric approach in future developments is crucial, focusing on minimizing radiation exposure, improving imaging comfort, and tailoring protocols to individual patient needs. Encouraging collaboration and data-sharing initiatives fosters robust research and validates AI models, contributing to PET-CT technology advancement. Evolving regulatory considerations for AI applications in medical imaging require ethical guidelines and frameworks for responsible deployment and patient safety. Continuous advancements in PET-CT, including TOF capabilities and AI-driven image reconstruction, promise more precise and efficient clinical assessments, enhancing patient care and outcomes. Addressing challenges like spatial resolution intricacies and motion artifacts is crucial for unlocking the full potential of PET-CT, paving the way for a paradigm shift in personalized and comprehensive healthcare.

### 7.1. Current Limitations

While PET-CT imaging has undergone significant advancements, it is essential to acknowledge and address existing challenges and limitations to ensure the continued progress and optimization of clinical utility. This discussion explores key areas of concern in PET-CT technology. Current limitations in PET-CT technology include challenges with the spatial resolution, motion artifacts, quantitative accuracy, radiation exposure, limited temporal resolution, lesion detection in certain tissues, cost, and availability of radiotracers. Achieving a balance between spatial resolution and sensitivity remains challenging, while motion artifacts and calibration inconsistencies affect the image quality and accuracy. Ensuring accurate quantitative measurements, minimizing radiation exposure, and improving lesion detection in challenging anatomical regions are ongoing concerns. Cost and accessibility barriers also limit widespread adoption. Addressing these limitations requires collaboration among researchers, clinicians, and industry stakeholders to drive ongoing research and technological innovations in PET-CT imaging.

### 7.2. Future Prospects

The ongoing research in PET-CT focuses on optimizing acquisition protocols, integrating AI for advanced analysis, and expanding clinical applications, promising enhanced capabilities and personalized patient management. Continuous refinement and exploration in hybrid imaging underscore the commitment to advancing medical technology, with innovations in reconstruction algorithms laying the foundation for a transformative impact. Future prospects include advancements in detector technologies, AI integration, theranostics, and expanded clinical applications, ensuring optimized patient care and driving the dynamic trajectory of PET-CT imaging toward innovation and patient-centered care.

**Advancements in Detector Technologies:** Advancements in detector technologies for PET-CT imaging promise significant enhancements in spatial resolution and sensitivity, potentially revolutionizing diagnostic capabilities by enabling the precise detection of smaller lesions. Historically limited by conventional detectors, ongoing research focuses on novel design approaches, leveraging advanced materials and computational methods to overcome these challenges.

One avenue of innovation revolves around the development of next-generation detector materials with superior properties conducive to enhanced imaging performance. By harnessing the unique characteristics of advanced materials, such as silicon photomultipliers (SiPMs), lutetium oxyorthosilicate (LSO), and cerium-doped lutetium-yttrium oxyorthosilicate (LYSO), researchers aim to overcome the limitations of traditional photomultiplier tubes (PMTs) and scintillation crystals. These materials offer improved photon detection efficiency, temporal resolution, and energy resolution, thereby facilitating the more accurate localization of annihilation events and enabling the detection of smaller lesions [144,145,146].

Researchers are exploring innovative detector geometries and configurations, such as three-dimensional (3D) detector arrays, to enhance the spatial resolution and sensitivity in PET-CT imaging. These advancements enable precise DOI measurement, mitigating errors and enhancing the spatial localization accuracy, especially in challenging clinical scenarios. Additionally, the integration of depth encoding techniques like TOF and CTR further improves the spatial localization precision.

Advancements in detector readout electronics and signal processing algorithms are crucial for maximizing information extraction from PET-CT scans. High-speed data acquisition systems and sophisticated signal processing algorithms enable real-time event processing and reconstruction, minimizing data loss and optimizing the image quality. Furthermore, ML algorithms such as convolutional neural networks (CNNs) and generative adversarial networks (GANs) offer novel approaches to enhance image reconstruction and denoising, improving spatial resolution and sensitivity [147].

Continued research and development efforts in detector technologies are expected to push the boundaries of spatial resolution and sensitivity in PET-CT imaging. These advancements hold promise for revolutionizing diagnostic precision, facilitating personalized treatment strategies, and enabling the detection of subtle anatomical abnormalities. Interdisciplinary collaboration and leveraging advances in materials science, electronics, and computational modeling are key to realizing transformative innovations in PET-CT imaging for improved medical diagnostics and patient care.

**Expanding Role of Artificial Intelligence:** AI is poised to transform PET-CT imaging, with sophisticated algorithms expected to revolutionize image reconstruction, interpretation, and diagnostic decision support. These advancements, coupled with the integration of explainable artificial intelligence (XAI), ensure transparency in the decision-making process, allowing clinicians to understand and trust the AI-generated results. AI algorithms will evolve to integrate diverse clinical data, enhancing holistic and personalized patient assessments. The evolving regulatory landscape in AI for PET-CT aims to ensure ethical deployment, emphasizing standardization in validation and implementation. These developments are crucial for widespread acceptance, enhancing patient care quality and efficiency [148,149,150].

**Theranostics and Targeted Therapy:** Theranostics, which integrates diagnostic imaging with targeted therapy, is poised to become increasingly prominent in medical practice. This approach utilizes PET-CT scans to visualize disease characteristics, enabling the development of personalized treatment strategies. By tailoring therapy to the specific features identified through imaging, theranostics has the potential to enhance treatment outcomes and minimize side effects. This convergence of diagnostic and therapeutic modalities represents a significant advancement in precision medicine, offering clinicians powerful tools to optimize patient care [151].

**Integration with Other Imaging Modalities:** The integration of PET-CT with complementary imaging modalities, including MRI and molecular imaging techniques, is poised to offer a comprehensive perspective on both anatomical structures and functional aspects. This seamless fusion of imaging modalities enhances diagnostic precision and provides a deeper understanding of disease mechanisms and progression. By combining multiple imaging techniques, clinicians gain valuable insights into the complex interplay between physiological and pathological processes, thereby advancing patient care and treatment strategies across various medical specialties.

**Diversification of Clinical Applications:** The scope of PET-CT applications extends beyond oncology, with anticipated expansion into various medical domains such as neurological disorders, cardiovascular diseases, and inflammatory conditions. This diversification enables the precise diagnosis, treatment planning, and monitoring of therapeutic responses across a spectrum of healthcare disciplines. As PET-CT technology evolves, its versatility in elucidating disease pathology and guiding clinical management is expected to contribute significantly to improved patient outcomes and comprehensive healthcare delivery.

**Development of Novel Radiotracers:** The development of novel radiotracers tailored to specific diseases and biological processes expands the scope of PET-CT applications, facilitating deeper insights into various pathologies and enabling clinicians to address a broader range of clinical scenarios. Additionally, innovations aimed at extending radiotracer half-lives promise improved availability and optimized imaging procedures, enhancing patient access to advanced diagnostics. Furthermore, advancements in molecular imaging techniques beyond traditional PET tracers offer targeted imaging approaches, aligning with precision medicine principles and promising to revolutionize diagnostic practices across medical specialties, ultimately improving patient outcomes [152,153].

**Technological Advancements for Accessibility:** Technological advancements in PET-CT imaging promise to enhance accessibility by introducing more portable and compact systems, which are particularly beneficial in remote or resource-limited areas. These innovations aim to bring advanced diagnostic capabilities closer to patients, facilitating timely and effective medical care. Portable PET-CT systems offer flexibility in imaging locations, including point-of-care settings, and can be deployed in mobile medical units, extending imaging services to underserved populations and remote communities.

**Patient-Centric Approach:** Future PET-CT developments prioritize a patient-centric approach, aiming to minimize radiation exposure, improve imaging comfort, and tailor protocols to individual needs, ensuring a balance between diagnostic efficacy and patient well-being. Ongoing research may reveal novel imaging biomarkers beyond traditional metrics, enhancing diagnostic assessments and patient care. Integrating patient-reported outcomes into PET-CT assessments offers valuable insights into disease impact and treatment effects, aligning with a patient-centered healthcare paradigm and aiming to improve overall patient experience and healthcare delivery.

**Integrating Augmented Reality:** The integration of augmented reality into PET-CT visualization may become a reality. AR technologies could provide clinicians with interactive, three-dimensional reconstructions during image interpretation, offering a more immersive and intuitive understanding of anatomical and functional information. This could revolutionize how practitioners interact with and derive insights from PET-CT data.

Moreover, the future of PET-CT imaging is characterized by a paradigm shift towards promoting collaboration and data sharing. Anticipated initiatives include the establishment of large-scale databases that aggregate imaging data from diverse populations. Such collaborative endeavors aim to facilitate robust research, expedite the validation of AI models, and foster the development of standardized imaging protocols. This collective approach not only enriches the knowledge base but also accelerates progress in PET-CT innovation, positioning it prominently at the forefront of medical imaging, with enduring implications for patient care worldwide.

## 8. Conclusions

In the realm of clinical imaging, the transformative journey of PET-CT stands as a testament to relentless innovation and its profound impact on diagnostic precision, therapeutic strategies, and patient care. As we conclude our exploration of the cutting-edge innovations in PET-CT multimodality imaging, it becomes evident that we are on the cusp of a new era in medical diagnostics.

The strides made in detector technologies, the integration of AI, and the evolution of hybrid imaging systems have collectively propelled PET-CT beyond its traditional boundaries, revolutionizing clinical practice. These advancements not only enhance our ability to detect and characterize diseases with unprecedented accuracy but also pave the way for personalized and targeted interventions, minimizing the impact of treatments on patients. The comprehensive review of clinical applications, from oncology to neurology and beyond, highlights the versatility of PET-CT in providing a holistic understanding of both anatomical and functional aspects of diseases. The emergence of novel PET-CT systems, the integration of AI for image interpretation, and the exploration of new imaging biomarkers signify a paradigm shift towards more patient-centric and precise healthcare.

Despite the remarkable progress, it is crucial to acknowledge the current limitations and challenges in PET-CT imaging technology. Motion artifacts, spatial resolution considerations, and the need for standardized quantitative measurements remain areas of active research and development. Addressing these challenges is integral to the ongoing commitment to refining and optimizing PET-CT for diverse clinical scenarios. Looking ahead, the future of PET-CT appears exceptionally promising. Anticipated developments, including advancements in detector technologies, AI augmentation, and the integration of emerging technologies, signal a trajectory marked by continuous evolution and refinement. The exploration of theranostics, novel radiotracers, and portable PET-CT systems further underscores the dynamic landscape that lies ahead.

As we stand at the forefront of this revolutionary era, it is imperative to emphasize the collaborative efforts of clinicians, scientists, engineers, and industry stakeholders. The synergy between these disciplines has been instrumental in propelling PET-CT to its current pinnacle and will remain indispensable for charting the future course. In conclusion, our journey through the landscape of PET-CT innovations is not just a reflection of technological advancements, it is a testament to the unwavering commitment to advancing patient care. The fusion of technological prowess, clinical insight, and a patient-centric approach positions PET-CT as a cornerstone in modern healthcare. This comprehensive review serves not only as a snapshot of the present state of PET-CT but also as a compass guiding us toward a future where clinical practice is revolutionized, one image at a time.

## Figures and Tables

**Table 1:** Summary of related survey articles.

Category	Essential Characteristic	Weaknesses (Compared to the Proposed Survey)
General tutorials Nicolas Aide et al., 2022 [19]; Omar Dzaye et al., 2023 [20]; Paul E. Kinahan et al., 2022 [21]	Explores PET-CT technology advancements, delving into the theory, clinical relevance, and prospects. Outlines the evolving role of PET in interventional oncology, highlighting its advantages in guiding interventions and evaluating treatment responses.	The article overlooks potential challenges and limitations of new PET-CT technologies, warranting a more critical examination of drawbacks in real-world clinical scenarios. Lacks an in-depth analysis of potential challenges or limitations associated with the integration of PET into clinical practice. Does not explore the existing solution by critically considering performance analysis.
Tutorials/survey M. Unterraineret et al., 2020 [22]; V. Bertolini et al., 2020 [23]; Johannes Schwenck et al., 2023 [24]	Addresses the lack of consistent guidelines for CT scans in PET-CT examinations. Conducts a systematic review to analyze CT protocols used in PET-CT, their purposes, and the correlation with dose optimization. All articles highlight recent innovations and advancements in PET-CT imaging technology, reflecting a focus on improving diagnostic precision and therapeutic outcomes. Acknowledges the interdisciplinary nature of PET-CT technology, and recognizing collaborations across radiology, nuclear medicine, and physics.	Lacking discussion about the practical challenges of integrating PET-CT innovations into routine clinical practice. Standards of care are lacking, hindering the assessment of PET-CT’s relative advantages. Insufficient evidence or discussion on how PET-CT advancements directly impact patient survival or quality of life.
Proposed Survey	Comprehensive coverage of recent advancements in PET-CT technology. Focus on emerging trends such as AI integration and hybrid imaging systems. Detailed analysis of technological innovations in PET detectors and CT scanners. Exploration of clinical applications across various medical disciplines. Critical examination of challenges and future directions in PET-CT imaging.	

**Table 2:** Summary of technological innovations in PET-CT.

Category	Article	Year	Achievements
PET Detectors	S. Surti et al., [25]	2020	The paper provides updates on TOF PET hardware, discussing scintillators, photosensors, electronics, detector designs, commercial scanners, challenges, and future sub-50 ps CTR.
	D.R. Schaart et al. [26]	2021	Outlines the evolution of clinical PET imaging, driven by advancements in TOF detectors, addressing challenges and future improvements.
	M.K. Singh et al., [28]	2024	Advancements in digital PET-CT include an optimized crystal size, enhanced TOF resolution, expanded axial field of view, and improved sensitivity.
	Z. Kuang et al., [36]	2023	Investigated DOI resolution impact on the spatial resolution in a SIAT aPET scanner. Found 2 DOI bins necessary for artifact-free images.
	Sánchez Gonzalo et al., [39]	2021	Improved PET scanner performance via fast, efficient photosensors and scintillators; research on segmented vs. monolithic crystal geometries and electronics optimization.
CT Scanner	Andersen et al., [46]	2020	Spectral dual-layer detector CT improves cancer detection and characterization, with increased sensitivity and decreased need for follow-up examinations. Spectral CT detected more cancers with a higher sensitivity (89%), but a slightly longer reading time (82 s), compared to conventional CT.
	G. Krokos et al., [49]	2021	Standardizing advanced reconstructions feasible; adjustments required for Gallium-68; significant differences in lesion SUVs between BPL and PSF reconstructions.
	J. Greffier et al., [42]	2023	Advancements in spectral CT, notably dual-energy and multi-energy systems, enhance tissue characterization, promising revolutionary diagnostic imaging and clinical applications.
	Q. Li et al., [44]	2020	A strong positive correlation was observed between the microvessel density (MVD) and iodine concentration (IC), highlighting the potential of spectral CT in lung cancer prognosis assessment.
Hybrid System	T. Shibutani et al., [53]	2023	Dual-isotope simultaneous acquisition (DISA) of 99mTc and 18F affects the 99mTc image quality due to crosstalk and spill-over. This study quantified these effects and evaluated correction strategies.
	Q. Zhang et al., [52]	2024	Multimodal AI techniques enhance pediatric total-body PET image quality, reducing the scan time without compromising the diagnostic accuracy, thus leveraging prior CT information.
	Kahvecioglu et al., [56]	2022	Evaluated the efficacy and toxicity of dose escalation with simultaneous integrated (SIB) or sequential boost (SB) for positive lymph nodes in locally advanced cervical cancer. Dose escalation with IMRT/VMAT achieved excellent local control for involved lymph nodes, with a low toxicity profile.
	J. Gong et al., [58]	2021	Compared the diagnostic performance of 18F-FDG PET-MRI versus PET-CT in the preoperative evaluation of cervical cancer, revealing PET-MRI’s superior specificity and sensitivity in delineating tumor boundaries and detecting bladder involvement, with elevated SUV values.
	A.M. Fowler [59]	2021	Compared glucose uptake measurements in invasive breast cancer using simultaneous PET-MRI and prone PET-CT, demonstrating strong correlation between modalities.

**Table 3:** Summary of image quality and quantification in PET-CT.

Category	Article	Year	Achievements
Noise-Reduction Techniques	I. Speck et al., [60]	2021	The study found significant background noise levels in modern air-cooled PET-CT scanners, impacting speech recognition, with implications for auditory pathway research.
	K. Weyts et al., [61]	2022	The study investigated the impact of AI-based denoising on halving the PET acquisition time in digital PET-CT, showing comparable image quality and lesion detection.
	Boughdad et al., [62]	2020	Post-reconstruction filters affected the prediction of the pathological complete response (pCR). The EANM images showed significant associations with pCR, suggesting potential impact on predictive accuracy of 18F-FDG PET-CT.
	H. Xie et al., [63]	2023	The Unified Noise-aware Network (UNN) addressed PET image noise variability, outperforming single-level networks, and offering better denoising for dynamic PET scans across diverse datasets.
	L. Xu et al., [64]	2022	Comparison of small voxel Bayesian penalized likelihood (SVB) and OSEM reconstructions for small lung lesion detection in phantom and patient studies showed SVB’s superiority without an increase in image noise.
	L. Fang et al., [65]	2024	Achieved a TOF resolution of 249 ps, a sensitivity of 22.1 cps kBq−1, and an NECR peak of 150.9 kcps. Demonstrated the ability to distinguish 2.0 mm rods in a phantom study and improved the SNR by 1.7 in human brain imaging with TOF.
Motion Correction	T. Kaji et al., [86]	2023	This study evaluated serial dynamic whole-body FDG PET imaging in 797 patients, finding motion artifacts in 13.3% of cases. Summing the images before body motion effectively minimized artifacts, improving the image quality.
	N. Miyaji et al., [87]	2023	MotionFree (AMF) with a data-driven respiratory gating (DDG) provided precise respiratory waveforms and improved the quantitative accuracy, which is particularly beneficial for patients with irregular breathing patterns.
	Yu-Jung Tsai et al., [88]	2023	The study explored motion correction in PET-CT using anatomical priors, aiming to align images and improve the reconstruction accuracy, showing promising preliminary results.
	B. Noto et al., [66]	2022	Respiratory motion correction in PET improved the lymph node assessment in lung cancer staging. Expert readers rated motion-corrected images higher, with significant changes in the SUV and MTV, and overall PET-CT performance.
Quantitative Accuracy	Shirakawa et al., [89]	2023	The SiPM-PET-CT evaluation highlighted iPSF’s minimal contrast impact but quantitative overcorrection, aiding in refining PET image reconstruction methods.
	Qing-Le Meng et al., [90]	2023	Motion-corrected image reconstruction (MCIR) enhanced tumor quantification and visual quality in PET-CT imaging by mitigating respiratory motion artifacts, with the DDG-based MCIR performing comparably to external device-driven methods.
	Zwezerijnen et al., [91]	2023	Variability in liver SUV measurements in lymphoma [18F]FDG PET-CT studies was reduced by using the robust SUVmeanliver metric and appropriate VOI sizes.
	E.E. Verwer et al., [92]	2021	The proposed harmonization criteria for PET brain studies aimed to ensure quantitative accuracy across diverse PET-CT systems, thereby enhancing the comparability in image quality.

**Table 5:** Benchmark datasets that are commonly used for evaluating PET-Ct multimodality imaging.

Benchmark Datasets	Purpose
NCI-ISBI 2013 Challenge	This dataset, provided by the National Cancer Institute (NCI) and the International Symposium on Biomedical Imaging (ISBI), includes PET-CT images of lung lesions for assessing computer-aided diagnosis algorithms.
RIDER Lung PET-CT	The Reference Image Database to Evaluate Therapy Response (RIDER) project provides a dataset of PET-CT scans of lung cancer patients before and after treatment, facilitating the evaluation of response assessment algorithms.
ADNI	The Alzheimer’s Disease Neuroimaging Initiative (ADNI) dataset includes PET-CT images of patients with AD and healthy controls, enabling the evaluation of PET-CT algorithms for neurodegenerative diseases.
NCI-MPTB	The NCI-Molecular PET-CT Dataset for Radiomics and AI Research contains PET-CT images of patients with head and neck cancer, providing a benchmark for developing radiomics and AI-based models.
ACRIN-FDG-PET-CT	The American College of Radiology Imaging Network (ACRIN) dataset consists of PET-CT scans acquired with FDG for various cancer types, facilitating research on tumor detection and characterization.
QIN-Head-Neck	The Quantitative Imaging Network (QIN) dataset includes PET-CT images of patients with head and neck cancer, allowing researchers to develop and validate quantitative imaging biomarkers.
Glioblastoma	This dataset comprises PET-CT scans of patients with glioblastoma, a type of brain tumor, and serves as a benchmark for studying the tumor metabolism and treatment response assessment.
KIT Whole-Body	The Karlsruhe Institute of Technology (KIT) provides a dataset of whole-body PET-CT scans for evaluating algorithms for lesion detection, segmentation, and classification in oncology imaging.
NCCBI	The National Cancer Center Biobank Imaging Dataset includes PET-CT images of various cancer types, facilitating research on tumor characterization, treatment response prediction, and outcome prediction.
NIST-Phantom	The National Institute of Standards and Technology (NIST) Phantom Dataset offers simulated PET-CT images of phantoms with known properties, enabling the evaluation of image reconstruction algorithms and quantitative analysis methods.

**Table 6:** Summary of AI-based advancements in PET-CT.

Article	Year	Target Area	Task	Achievements
Zaharchuk et al., [123]	2021	Oncology, Cardiology, Neurology	*ANNs for segmentation, detection, and classification.	AI enhanced PET-CT and PET-MRI, improving the image quality and safety, necessitating collaboration among researchers and physicians for innovative patient care.
M. Hatt et al., [124]	2021	Radiomics	Automating radiomics with GANs.	AI, especially deep learning, automated tasks, enhanced accuracy, and unlocked insights in PET-CT radiomics, revolutionizing imaging analysis.
P. Borrelli et al., [125]	2021	Lung Lesions	AI/dual *CNNs for lesion detection.	AI detected lesions with a 90% sensitivity, missing one small lesion. A strong correlation (R2 = 0.74) and a high agreement.
Sachpekidis et al., [126]	2023	Lymphoma	AI/CNN and lymphoma segmentation.	DL segmentation accurately predicted *TMTV, validating its prognostic utility for progression-free and overall survival in lymphoma patients.
Sadaghiani et al., [127]	2021	Cancer (Multiple Organs)	AI/ML segmentation, staging, resp. assessment, and prognosis.	AI enhanced the 18F-FDG PET imaging for cancer diagnosis and management by assisting in the detection, segmentation, response assessment, prognosis prediction, and improving image quality through ML algorithms.
Schwyzer et al., [128]	2020	Lung	AI/ML and pulmonary nodules detection.	Improved the detection of small pulmonary nodules in PET-CT scans using a DL algorithm with *BSREM reconstruction. The AI *AUC was higher with BSREM (0.848) than OSEM (0.796). A higher sensitivity with BSREM.
J. Wang et al., [78]	2024	Neurology	AI-assisted Parkinson’s diagnosis.	AI-assisted image analyses were highly accurate in diagnosing Parkinson’s, and distinguishing PD from normal controls and atypical parkinsonism.
L. Wei et al., [129]	2021	Oncology	AI/DL tumor detection and segmentation.	DL outperformed traditional methods in PET-CT response evaluation. CNN predicted the therapy response accurately. AI aided in patient identification and cancer detection.
Zirakchian et al., [79]	2022	Liver Tumor	CNNs, detection, and contour segmentation.	The addition of PET to CT enhanced the *CLM detection, margin assessment, and real-time visualization during ablation, reducing the need for contrast.
Veziroglu et al., [130]	2023	Lymphatic	AI, 3D UNet, lymphoma segmentation, and classification.	The AI model achieved a 0.88 median *DSC for lymphoma segmentation, correlating with manual segmentation and prognosticating progression-free and overall survival.

* Key terms: Artificial Neural Network (ANN); Convolutional Neural Network (CNN); Total Metabolic Tumor Volume (TMTV); Area Under the Curve (AUC); Block Sequential Regularized Expectation Maximization (BSREM); Colorectal Liver Metastasis (CLM); Dice Similarity Coefficient (DSC).

## Data Availability

The research data supporting this systematic review are from previously reported studies and datasets, which have been cited. The processed data are available from the corresponding author upon request.
